# Pink Pigmented Facultative Methylotrophic Bacteria Isolated from Fermented Philippine Shrimp Paste

**DOI:** 10.21315/tlsr2021.32.2.10

**Published:** 2021-06-29

**Authors:** Christian Jordan O. dela Rosa, Anthony C. Lee, Windell L. Rivera

**Affiliations:** 1 Pathogen-Host-Environment Interactions Research Laboratory, Institute of Biology, College of Science, University of the Philippines Diliman, Quezon City 1101, Philippines; 2 Biology Department, College of Science, De La Salle University, City of Manila 0922, Philippines

**Keywords:** Pink Pigmented Facultative Methylotrophic Bacteria, *Methylobacterium*, Shrimp Paste, 16S rRNA Gene, Philippines

## Abstract

Pink pigmented facultative methylotrophic (PPFM) bacteria are ecologically distributed microorganisms. They have been isolated in many types of ecosystems like soil, water, air, in association with plants and even as pathogens in humans. However, a yet unexplored area for PPFM bacteria research is in food. Hence, the objective of this study was to establish the presence of PPFM bacteria in Philippine fermented food, in particular shrimp paste, and characterise them phenotypically and genotypically. A total of 13 PPFM bacteria were obtained from Philippine shrimp paste. Sequencing of the 16S rRNA gene revealed that the PPFM bacterial isolates belong to the genus *Methylobacterium*. A total of 35 phenotypic characterisations were performed that included morphological, biochemical and physiological tests. Phylogenetic tree was constructed to establish the genetic relatedness of the isolates. Morphological test results showed that all 13 isolates were consistent with the established phenotypic characters of the genus such as pink colony colour, Gram negative and rod-shaped. Biochemically, the use of API® 20 NE tests showed heterogeneity of results and physiological tests exhibited that the isolates are primarily mesophilic and halotolerant, being able to grow at 2% salt. Phylogenetic analysis showed that the isolates are *Methylobacterium populi, M. dankookense, M. lusitanum, M. radiotolerans* and *M. zatmanii*. This study confirmed the presence and diversity of PPFM bacteria in Philippine shrimp paste. Further studies are needed to show the functional activity of *Methylobacterium* in Philippine shrimp paste production.

**Highlights**

- This study is the first to identify *Methylobacterium* spp. from Philippine shrimp paste using 16S rRNA gene sequencing.
- The 13 *Methylobacterium* isolates belong to five different species which are: *M. populi, M. dankookense, M. radiotolerans, M. lusitanum* and *M. zatmanii*.
- Of the five species, three were previously reported to be pathogenic and resistant to some known antibiotics.

## INTRODUCTION

Fermentation is one of the oldest forms of food preservation technology in the world (Aloys & Angeline 2009). It constitutes an essential contribution to human diets in many countries because it is an inexpensive way of preserving and improving food nutritional value while enhancing sensory properties (Gadaga *et al*. 1999). Microbiologically, it is the desirable process of biochemical modification of food products brought about by microorganisms and their enzymes that may lead to the detoxification and destruction of undesirable factors present in raw food like tannins and polyphenols. Some microorganisms may participate in parallel process of fermentation, while others act in a sequential manner with a changing dominant flora during the course of fermentation (Gadaga *et al*. 1999; Aloys & Angeline 2009).

The Philippines has produced several fermented products like *nata de coco* (coconut gel), *buro* (fermented rice with fish), *puto* (acid-fermented leavened bread) and *atchara* (fermented pickled *Carica*), among others. Some of these products are even exported to other countries such as shrimp paste or more vernacularly known as *bago-ong* that constitutes 42% of the total Philippine exported processed food. Shrimp paste is considered as a common condiment in Filipino diet because it serves as a flavouring agent in many Philippine dishes (Montaño *et al*. 2001). Additionally, it provides consumers with essential nutrients like fats, carbohydrates, fiber, calcium, phosphorus, iron, *β*-carotene, vitamin A, thiamine, riboflavin, niacin and ascorbic acid. Shrimp paste is a relatively common cuisine in other Asian countries like Korea (*jeotgal*), Indonesia *(terasi*) and Thailand *(kapi*). However, just like other fermented food products, the microbiology of shrimp paste is quite complex and largely unknown (Blandino *et al*. 2003).

In most studies, lactic acid bacteria (LAB) and those belonging to *Bacillus* species are the dominant microflora in shrimp paste. LAB are responsible for keeping the quality, safety and extended shelf-life of the food due to its ability to lower the pH, produce ethanol, hydrogen peroxide and bacteriocins (Yang *et al*. 2014; Mokoena 2017). On the other hand, *Bacillus* species produce and secrete large quantities of extracellular enzymes for the breakdown of proteins (Schallmey *et al*. 2004). Interestingly, pink pigmented facultative methylotrophic (PPFM) bacteria have been isolated from fermented foods such as *Methylobacterium jeotgali* from *jeotgal* and *M. extorquens* from *kapi* (Aslam *et al*. 2007; Tapingkae *et al*. 2010).

PPFM bacteria are an interesting group of prokaryotic eubacteria. They are phylogenetically diverse and ubiquitous due to their capacity to grow in reduced carbon substrates, or in a wide range of multi-carbon substrates as carbon source (Balachandar *et al*. 2008; Vuilleumier *et al*. 2009). Thus, they play an essential role in the earth’s carbon cycling by means of their participation in methane oxidation and C1 metabolism (Wang *et al*. 2004). PPFM bacteria are pink in colour because of the presence of carotenoids rendering them tolerant to extreme light conditions and radiations (Kumar & Lee 2009). These bacteria have been isolated in air (Lee & Jeon 2018), soil (Madhaiyan *et al*. 2007), water (Gallego *et al*. 2005), plants (Kumar & Lee 2009) and humans (Hornei *et al*. 1999).

This present study is relevant since some PPFM bacteria have been documented to be opportunistic (Kovaleva *et al*. 2014) and antibiotic-resistant that have caused bacteremia (Lai *et al*. 2011; Hornei *et al*. 1999; Truant *et al*. 1998) and chronic granulomatous disease (Liana Falcone *et al*. 2016). Microbial populations and their potential interactions are highly significant to ensure that food production is safe and of high-quality (Justé *et al*. 2008). The presence of PPFM bacteria in fermented food especially if they are known pathogens such as *M. radiotolerans, M. zatmanii* and *M. lusitanum* will have great impact on food safety. Microbial diversity study of Philippine shrimp paste dealing with PPFM bacteria is significant since shrimp paste production in many regions in the Philippines is being done through backyard small-scale industries. Thus, the general objective of this study was to establish the presence of PPFM bacteria from Philippine-produced shrimp paste. Specifically, it aimed to describe and identify the isolated PPFM bacteria using standard culture methods and 16S rRNA gene sequence analysis.

## MATERIALS AND METHODS

### Isolation of PPFM Bacteria from Philippine-Made Shrimp Paste

Two Philippine-made shrimp paste samples were taken from two distant places outside of Metro Manila, namely Pangasinan and Bulacan provinces. From each location, three sub-samples were taken either from the local wet market or directly from local manufacturers doing small-scale backyard shrimp paste production. Shrimp paste sampling collections were done on the same day and kept in an ice box during transport to the laboratory and were immediately refrigerated at 4°C upon arrival.

After 24 h, an enrichment culture was prepared by adding 10 g of the shrimp paste samples onto a 90-mL sterile ammonium mineral salts (AMS) + 0.5% methanol (Woo *et al*. 2012). The enrichment set-up was incubated for 4 days at 30°C. Afterwards, decimal serial dilution was done by adding 1 mL of the enrichment suspension to 9 mL of peptone water. 0.1 mL aliquots of the last three dilutions were spread plated in duplicates using AMS agar + 0.5% methanol. Plates were incubated for 4 days at 30°C.

Distinct pink colonies were selected and re-streaked onto fresh AMS agar + 0.5 methanol for purification and incubated at 30°C for 4 days. Initially, the isolates were grown in a selective medium (AMS + 0.5% methanol) that allowed the growth of *Methylobacterium* and inhibited the growth of other microorganisms. To ensure that an isolate was pure, it was re-streaked on a less selective medium, glycerol-peptone agar (GPA: 10 g glycerol, 10 g peptone, and 15 g agar in 1 L distilled water). If there were inhibited microorganisms, then they would grow in GPA; if none, it means that the isolates were already pure cultures. The clock streak method was performed to get well-isolated colonies. Colonies were streaked on GPA for maintenance. Subculture was done every 3 weeks.

### Phenotypic Characterisation

Pure cultures of isolates were subjected to morphological, biochemical and physiological tests. Gram staining and special staining for polyhydroxybutyrate (PHB) inclusion bodies were done to determine cellular characteristics such as Gram reaction, shape and presence of PHB granules.

Biochemical tests included catalase, oxidase, and API® 20 NE (bioMerieux, France). The API® 20 NE was used not for identification but purely for biochemical characterisation due to the limited database it possesses which only included *M. mesophilicum* out of the 50 taxonomically identified and valid species of *Methylobacterium*. Results of the API® 20 NE were referred to the manufacturer’s guide.

Isolates were also subjected to different physiological parameters such as incubation in varying temperatures (4°C, 30°C, 37°C and 56°C), reduced carbon compounds such as carbon source other than methanol (0.5% formaldehyde and 0.5% chloroform), which were added to AMS agar as the basal medium and incubated at 30°C with differing salt concentrations (0, 2%, 7%, 14% and 20% NaCl). Tests for salt concentrations were done using GPA as basal medium and incubated at 30°C. All tests were done in duplicates.

### Molecular Identification and Phylogenetic Analysis

DNA extraction was done using the protocol described by InstaGene Matrix™ (Bio-Rad Laboratories, USA). Briefly, 1 mL of PPFM bacteria grown in AMS + 0.5% broth was suspended in a microfuge tube and centrifuged twice at 13,000 g for 3 min. The pellet was washed by adding 1 mL of sterile, distilled, deionised (sdd) water and recentrifuged for 1 min at 12,000 rpm. A 50 μL aliquot of the InstaGene Matrix™ was added to the pellet and incubated at 56°C for 20 min. Afterwards, the tube was vortexed at high speed for 10 s and placed in a boiling water bath for 8 min. Lastly, the aliquot was vortexed for 10 s, centrifuged for 2 min at 12,000 rpm, and stored at −20°C until further use.

Genomic DNA was amplified using KAPATaq DNA polymerase using 16S rRNA gene-specific primers (Forward 63f: 5′ CAG GCC TAA CAC ATG CAA GTC 3′; Reverse 1387r: 5′ GGG CGG WGT GTA CAA GGC 3′) (Marchesi *et al*. 1998). The cycling conditions were as follows: 94°C for 2 min, followed by 30 cycles of 94°C for 30 s, 50°C for 1 min and 45 s, and 72°C for 1 min and 45 s, and final extension of 72°C for 5 min. PCR products were separated using 1% (w/v) agarose gel electrophoresis and visualised by staining with SYBR Safe DNA gel stain (Invitrogen) and UV illumination. PCR products were sent to First BASE Laboratories (Selangor, Malaysia) for DNA purification and sequencing. Acquired chromatograms were aligned using Basic Local Alignment Search Tool (BLAST). Aligned sequences were cross-referenced to NCBI database for identification (https://blast.ncbi.nlm.nih.gov/Blast.cgi). Sequences were then deposited to GenBank.

Phylogenetic tree was constructed using the maximum likelihood (ML) method, and evolutionary distances were computed using Tamura-Nei method. A bootstrap confidence analysis was performed on 1,000 replicates to determine the reliability of the tree topology. Bootstrap values below 60% were considered invalid.

## RESULTS

### PPFM Bacteria from Philippine Shrimp Paste

**Figure 1:**
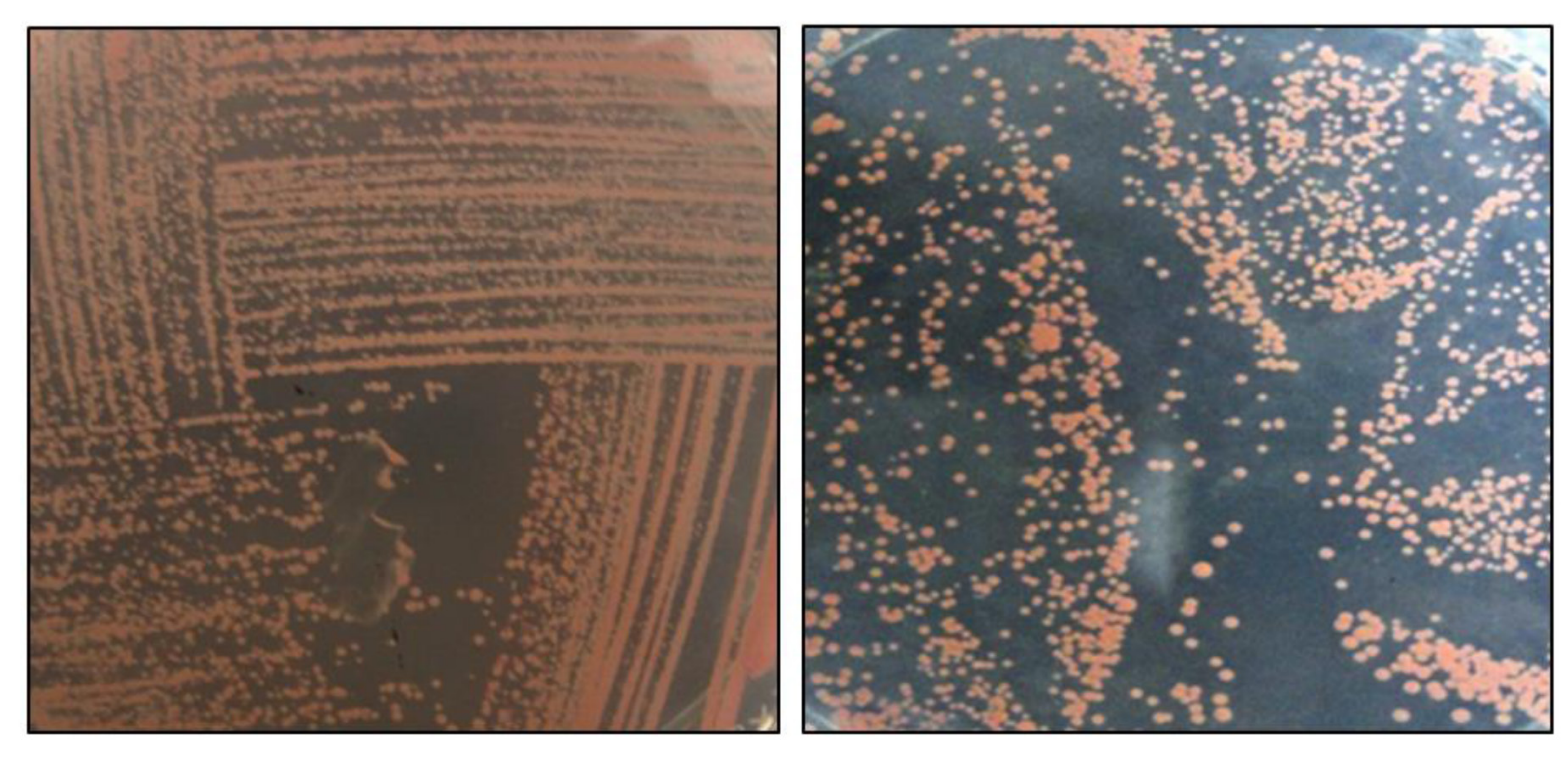
Pink colonies of PPFM bacteria grown in AMS agar supplemented with 0.5% methanol.

PPFM bacteria are slow growing microorganisms that start to have visible pink growth at AMS + 0.5% methanol only after 3 to 4 days of incubation at 30°C as shown in Fig. 1. Visible pink colonies of differing intensity served as the primary basis for isolation.

All isolates were facultative methylotrophic as they have initially shown to grow in a medium with reduced carbon source (AMS + 0.5% methanol) followed by a multi-carbon medium using GPA for purification. A total of 13 isolates were identified as *Methylobacterium* by subjecting the reconstructed DNA sequences composed of 900 kb to 1200 kb using BLAST to reference the closest possible match through high percent similarity and high query coverage. Of the 13 *Methylobacterium*, six were specifically identified as *M. populi* (JX993406, JX993409, JX993410, JX993428, JX993429, JX993432), four *M. radiotolerans* (JX993420, JX993425, JX993427, JX993431), one *M. dankookense* (JX993414), one *M. lusitanum* (JX993418), and one *M. zatmanii* (JX993430). All the isolates yielded a 95% to 99% similarity with sequences deposited in GenBank.

### Phenotypic Characterisation

A total of 35 phenotypic tests were performed with three morphological characterisations, 22 biochemical tests and 10 physiological tests as shown in Table 1. PPFM bacterial colonies ranged from light pink to pinkish orange. Cells were stained Gram negative, rod-shaped and shown to produce PHB granules. All isolates were aerobic and catalase- and oxidase-positive. They were shown to be incapable of utilising potassium nitrate, L-tryptophan, L-glucose (for acidification), L-arginine, esculin, gelatin (bovine origin), 4-nitrophenyl-β-D-galactopyranoside, caprate, adipate and phenylacetate. On the other hand, isolates exhibited variations in the utilisation of urea, L-glucose (assimilation), L-arabinose, L-mannose, L-mannitol, N-acetyl-glucosamine, L-maltose, malate and citrate. All isolates were capable of growth in mesophilic conditions, and at 2% NaCl. All isolates were also capable of growing in toxic conditions such as in formaldehyde and chloroform.

### Phylogenetic Analysis

**Figure 2:**
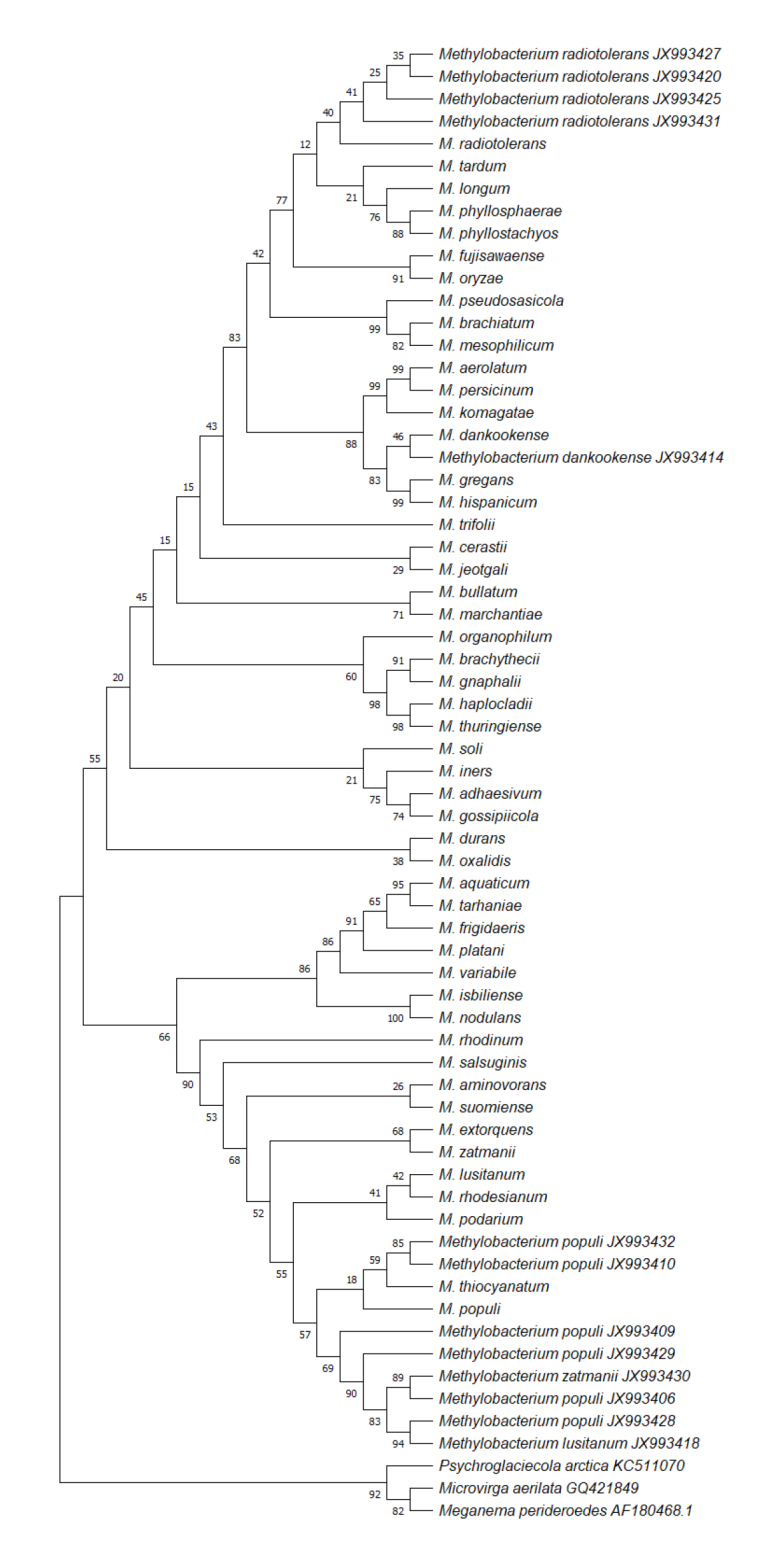
Maximum likelihood (ML) tree based on the Tamura-Nei model with 1,000 replicates of the 13 *Methylobacterium* sp. isolates from Philippine shrimp paste inferred using a comparative analysis of 16S rRNA gene sequences with 50 valid type specimens of *Methylobacterium* and rooted with the outgroups, *Microvirga aerilata* (GQ421849.1), *Meganema perideroedes* (AF180468.1) and *Psychroglaciecola arctica* (KC511070.1).

Sequences of PPFM bacterial isolates were aligned together with the sequences of 50 valid species of *Methylobacterium* using MEGA-10 to create a phylogenetic tree with *Microvirga aerilata, Meganema perideroedes* and *Psychroglaciecola arctica* (all belong to the same family of Methylobacteriaceae) serving as outgroups as shown in Fig. 2 (http://www.bacterio.net/). Based on the tree, *M. dankookense* (JX993414) clustered with the *M. dankookense* type specimen. Also, all four *M. radiotolerans* clustered with the *M. radiotolerans* type specimen. Of the eight *M. populi*, two clustered with the main branch covering the *M. populi* type specimen. On the other hand, the remaining six *Methylobacterium* formed a polyphyletic group with *M. populi* type specimen.

## DISCUSSION

PPFM bacteria have been documented worldwide from different habitats ranging from non-living environments (soil, water, air) or in association with living things (plants and humans) (Omer *et al*. 2004). In the Philippines, research on PPFM bacteria is inadequate, if not in a standstill. One generally not much explored area of study for PPFM bacteria is in food specifically in fermented foods since the process is primarily microbiological. So far, there is only a handful of research that focuses primarily on PPFM bacteria in fermented foods (Aslam *et al*. 2007; Tapingkae *et al*. 2010).

In this study, all 13 isolates were shown to be methylotrophic since they grew in carbon-reduced medium (AMS + 0.5% methanol). In order to determine if the isolates were either obligate or facultative, they were grown in GPA, a medium that contains three carbon molecules. Microorganisms capable of growing in reduced carbon source, and that uses methanol as the sole carbon source are methylotrophic. On the other hand, methylotrophic microorganisms capable of growth in a medium with more than one carbon source are known to be facultative methylotrophic (Bratina *et al*. 1992; Taguchi *et al*. 1997). The 13 PPFM bacterial isolates were molecularly identified and were phylogenetically related to the genus *Methylobacterium*. Molecular identification showed that they are *M. populi, M. radiotolerans, M. dankookense, M. lusitanum* and *M. zatmanii*.

Eight isolates were phylogenetically related to the type specimen described to be associated with plants, *M. populi*, an endophyte of the Poplar tree (*Populus deltoides* x *nigra* DN34) (Van Aken *et al*. 2004). This is because *Methylobacterium* has the ability to utilise plant-derived methanol as energy source, hence, allowing them to colonise plants in large numbers (Sy *et al*. 2005; Abanda-Nkpwatt *et al*. 2006; Delmotte *et al*. 2009). Others have been described to be isolated in water such as *M. dankookense* (Lee *et al*. 2010), *M. tardum* (Kato *et al*. 2008), and *M. fujisawaense* (Green *et al*. 1988). These show the cosmopolitan nature of *Methylobacterium* and also the microbial diversity of Philippine shrimp paste. It is interesting to note that *Methylobacterium* spp. are present in fermented foods given that they are aerobic bacteria and fermentation is an anaerobic process. Further research is recommended to establish the physiological mechanism of *Methylobacterium* in an anaerobic condition and also its role in shrimp paste production.

Phenotypically, isolates were consistent with the description of *Methylobacterium* as pink-producing colonies, Gram negative and produce PHB granules (Taguchi *et al*. 1997; Singh & Parmar 2011). The pink colouration is due to the natural ability of *Methylobacterium* for the production of carotenoids such as astaxanthin, zeaxanthin and canthaxantin (Van Dien *et al*. 2003; Stepnowski *et al*. 2004). Notably, the pinkish colour distinct in Philippine shrimp paste is mostly associated with the astaxanthin from shrimps (Sowmya *et al*. 2017), however, with the presence of *Methylobacterium* in Philippine shrimp paste, it may be hypothesised that *Methylobacterium* may contribute to the overall nutritive value of Philippine shrimp paste. Additionally, *Methylobacterium* spp. have been shown to be resistant to many environmental stresses such as dehydration, chlorination, ionization, freezing and exposure to toxic compounds, like in this study wherein the isolates were capable of growing in chloroform (Goodwin *et al*. 2001; McDonald *et al*. 2001; Trotsenko *et al*. 2001).

However, an even more important aspect beyond PPFM’s role in shrimp paste production is that *Methylobacterium* has been shown to be an opportunistic pathogen (Kovaleva *et al*. 2014). As a matter of fact, of the five species of *Methylobacterium* in this study, three have been reported in literature to be pathogenic. *M. radiotolerans* and *M. zatmanii* have been reported to cause severe bacteremia (Truant *et al*. 1998; Hornei *et al*. 1999; Lai *et al*. 2011). *M. lusitanum* has been isolated from patients with chronic granulomatous disease (Liana Falcone *et al*. 2016). Moreover, the three *Methylobacterium* mentioned have been found to exhibit resistance to many kinds of antibiotics such as meropenem, ciprofloxacin, co-trimoxazole, ceftazidime, ceftizoxime and ceftriaxone (Truant *et al*. 1998; Hornei *et al*. 1999). Therefore, having isolated *Methylobacterium* from shrimp paste, a common condiment in the Philippines, would impact food safety and surveillance. Although *M. populi* and *M. dankookense* are not pathogenic, horizontal gene transfer between them may happen, increasing the risk factor.

Taxonomically, due to the limited database and with the chemotaxonomic heterogeneity of *Methylobacterium*, isolates were subjected to phylogenetic analysis to show genetic relatedness. *M. populi* isolates (JX993406, JX993409, JX993410, JX993428, JX993429, JX993432) are shown to be genetically related to *M. populi* type specimen matching molecular identification. The same goes with JX993414 as *M. dankookense* and all four isolates molecularly identified in NCBI as *M. radiotolerans* did form a monophyletic group with *M. radiotolerans* type specimen further confirming their identities. Lastly, *M. lusitanum* and *M. zatmanii* are phylogenetically more related with *M. populi* than their respective type specimens. This shows the problematic taxonomy of *Methylobacterium*, even more, if we are going to rely on one gene marker (16S rDNA). Thus, it is recommended that housekeeping genes (*gyrB, atpD, dnaK, glnl, recA, rpoB*) and chemotaxonomic differences to back up 16S rDNA in identifying PPFM bacteria (Green & Ardley 2018).

## CONCLUSION

In conclusion, this study provided an additional insight on the microbial composition and diversity of Philippine shrimp paste. To the authors’ knowledge, this is the first study that identified species of *Methylobacterium* from Philippine shrimp paste. The microbiological safety of shrimp paste production only checks for typical food pathogens such as *Escherichia coli* but not *Methylobacterium*. The researchers believe that the presence of *Methylobacterium* should be included since known pathogenic species that cause bacteremia and reported to be antibiotic-resistant have been isolated from this study. This research with its new findings can better improve food quality and surveillance in Philippine shrimp paste production. Lastly, it is recommended that future analyses should be done to determine the functional influence of *Methylobacterium* to the overall Philippine shrimp paste production and also its influence with respect to other microorganisms that constitute the microbial community of Philippine shrimp paste.

## Figures and Tables

**Table 1:** Phenotypic characteristics of PPFM bacteria from Philippine shrimp paste.

Characteristic	JX9934-												

	06	09	10	14	18	20	25	27	28	29	30	31	32
Bacterial shape	rod	rod	rod	rod	rod	rod	rod	rod	rod	rod	rod	rod	rod
Gram reaction	−	−	−	−	−	−	−	−	−	−	−	−	−
Presence of PHB	+	+	+	+	+	+	+	+	+	+	+	+	+
Catalase	+	+	+	+	+	+	+	+	+	+	+	+	+
Oxidase	+	+	+	+	+	+	+	+	+	+	+	+	+
Substrate utilisation:													
Potassium nitrate	−	−	−	−	−	−	−	−	−	−	−	−	−
L-tryptophan	−	−	−	−	−	−	−	−	−	−	−	−	−
L-glucose	−	−	−	−	−	−	−	−	−	−	−	−	−
(acidification)													
L-arginine	−	−	−	−	−	−	−	−	−	−	−	−	−
Urea	−	−	−	+	+	+	+	+	−	−	+	+	−
Esculin	−	−	−	−	−	−	−	−	−	−	−	−	−
Gelatin (Bovine origin)	−	−	−	−	−	−	−	−	−	−	−	−	−
4-nitrophenyl-β-D-galactopyranoside	−	−	−	−	−	−	−	−	−	−	−	−	−
L-glucose (assimilation)	+	+	+	−	−	−	−	−	+	+	−	−	+
L-arabinose	+	+	+	−	−	+	+	+	+	+	−	+	+
L-mannose	+	+	+	−	−	−	−	−	+	+	−	−	+
L-mannitol	+	+	+	−	−	−	−	−	+	+	−	−	+
N-acetyl-glucosamine	−	−	−	−	+	−	−	−	−	−	−	−	−
L-maltose	+	+	+	−	−	−	−	−	+	+	−	−	+
Gluconate	−	−	−	+	−	+	+	+	−	−	−	+	−
Caprate	−	−	−	−	−	−	−	−	−	−	−	−	−
Adipate	−	−	−	−	−	−	−	−	−	−	−	−	−
Malate	−	−	−	+	+	+	+	+	−	−	+	+	−
Citrate	−	−	−	+	−	−	−	−	−	−	−	−	−
Phenylacetate	−	−	−	−	−	−	−	−	−	−	−	−	−
Growth at:													
4°C	+	+	+	−	−	−	−	−	+	+	+	−	+
30°C	+	+	+	+	+	+	+	+	+	+	+	+	+
37°C	+	+	+	−	+	+	+	+	+	+	+	+	+
56°C	−	−	−	−	−	−	−	−	−	−	−	−	−
2% NaCl	+	+	+	+	+	+	+	+	+	+	+	+	+
7% NaCl	+	+	+	−	−	−	−	−	+	+	−	−	+
14% NaCl	−	−	−	−	−	−	−	−	−	−	−	−	−
20% NaCl	−	−	−	−	−	−	−	−	−	−	−	−	−
Carbon source:	+	+	+	+	+	+	+	+	+	+	+	+	+
0.5% formaldehyde													
Carbon source: 0.5% chloroform	+	+	+	+	+	+	+	+	+	+	+	+	+

*Note*: (+) Positive; (−) negative
